# Clinical characteristics of 662 patients with pancreatic neuroendocrine tumors receiving antitumoral therapy

**DOI:** 10.1097/MD.0000000000032044

**Published:** 2022-12-16

**Authors:** Sven H. Loosen, Karel Kostev, Johannes Eschrich, Sarah Krieg, Andreas Krieg, Tom Luedde, Henning Jann, Christoph Roderburg

**Affiliations:** a Clinic for Gastroenterology, Hepatology and Infectious Diseases, University Hospital Düsseldorf, Medical Faculty of Heinrich Heine University Düsseldorf, Düsseldorf, Germany; b Epidemiology, IQVIA, Frankfurt, Germany; c Department of Hepatology and Gastroenterology, Charité University Medicine Berlin, Berlin, Germany; d Department of Surgery (A), University Hospital Duesseldorf, Heinrich-Heine-University Duesseldorf, Duesseldorf, Germany

**Keywords:** NET, pancreas, pNET, SSA

## Abstract

Pancreatic neuroendocrine neoplasia constitute an important subentity of the gastroenteropancreatic neuroendocrine neoplasms accounting for up to 15% of all neuroendocrine neoplasm. Prognosis and oncological behavior of pancreatic neuroendocrine tumors (pNETs) is extremely heterogenous and dependent on the specific tumor stage and differentiation. However, systematic data on the specific epidemiology of pNET are scarce. We identified 662 patients with pNET within the Oncology Dynamics database (IQVIA). Patients were derived from 4 European countries (Germany, France, UK, Spain), 3 Asian countries (Japan, China, South Korea) and 2 South American countries (Mexico and Brazil) and with regard to major patient and tumor related characteristics including patients’ age, sex, tumor stage, tumor grading, and differentiation. The mean age of the study cohort was 62 years (SD 12 years) with 53.9.1% of all patients being male. The majority of patients had an Eastern co-operative of Oncology Group 1 performance status (63.3%). The most common Union international contre le cancer tumor stage was stage IV (85%) with liver metastases (89.0%) representing the most common site of extra-pancreatic tumor manifestation. The majority of all patients displayed well or moderate tumor differentiation (9.6% of patients had a Ki-67 expression below 2%. 67.6% of pNET patients had a Ki-67 expression between 2 and 20% and 22.8% of patients showed an expression above 20%). At time point of diagnoses, 93.1% of patients were classified as inoperable. Of note, 93.9 % of patients received systemic anti-tumoral therapy in palliative intention, while treatment was administered in 1.4 % of cases in neoadjuvant and in 4.7% of cases in in an adjuvant setting. Biological therapy was applied to 39.4% of patients, followed by targeted therapies (31.4%) and chemotherapy. Pancreatic neuroendocrine neoplasia are diagnosed in advanced tumor stages, globally. Systemic treatment was the most commonly used treatment modality. Such data may help to better understand the specific epidemiology of pNET worldwide.

## 1. Introduction

Neuroendocrine neoplasms (NEN) are defined as a heterogeneous family of malignant tumors derived from the disseminated neuroendocrine system that can be found almost anywhere in the human body but are most commonly located in the gastrointestinal tract and pancreas.^[1]^ Gastroenteropancreatic neuroendocrine neoplasms (GEP-NEN) account for approximately two-thirds of all NEN cases. Gastroenteropancreatic neuroendocrine are further subdivided according to the specific tumor localization with pancreatic neuroendocrine tumors (pNET) often exhibiting a more aggressive behavior than other GEP-NEN. Apart from their site of origin, NEN also show a wide variety in tumor morphology, differentiation status and proliferation. The current WHO classification system is organized by the tumor’s growth rate (with a Ki-67 fraction of < 3% representing grade 1 (G1), 3–20% grade 2 (G2), and > 20% grade 3 (G3)) and also discriminates between well-differentiated tumors and poorly differentiated carcinomas.^[2]^ Whereas low-grade NEN are malignancies with a near to normal life expectancy,^[3]^ the clinical management of high-grade NEN remains challenging. In contrast to their slow growing counterparts, highly proliferative G2 and G3 NEN are more aggressive, often diagnosed in advanced stages that are not amenable to curative surgery^[3]^ and therefore require systemic treatments.

Even though the incidence of pNET has been increasing in the last 3 decades,^[3]^ the general case numbers are still low. We and others have recently demonstrated that regional variations in the distribution of GEP-NEN are high, although the data are very limited.^[4]^ Here, we analyzed the general epidemiology of pNET in 4 European countries (Germany, France, UK, and Spain), 3 Asian countries (Japan, China, South Korea) and 2 South American countries (Mexico, Brazil) by using the Oncology Dynamics database (IQVIA). Such data may help to better understand the characteristics of patients with pNET in very different countries with sometimes very different health care systems and diagnostic or therapeutic possibilities.

## 2. Methods

### 2.1. Database

This retrospective cross-sectional study is based on the data from IQVIA’s Oncology Dynamics (OD) database^[5–7]^. This source is a cross-sectional partially retrospective survey collecting anonymized patient cases from a representative panel of oncologists in several countries worldwide. Data collection and reporting is conducted through a standardized online questionnaire where all items are mandatory. Missing data might occur if specific items were not available (e.g. Ki-67). Physicians are also asked to enter factual information from the patient medical record to avoid recall bias. Further tactics to ensure input accuracy include controlled code lists and multiple-choice questions, as well as interactive filters that limit nonapplicable questions. Physicians are instructed to report the most recent consecutive cases (up to 20 cases depending on the specialty) they had treated during the last 7-day period to discourage selective case submission. After the form submission, additional validations and trend checks are performed; anomalous values are discussed with the submitting participant and corrected as needed. OD database is considered representative as patient distributions are similar to those documented in the published literature.^[8]^

### 2.2. Patient selection and study variables

Surveys of all patients with pNET between January 1^st^ 2017 and March 31^st^ 2022 were available for 4 European countries (Germany, France, UK, Spain), 3 Asian countries (Japan, *China*, South Korea), and 2 South American countries (Mexico, Brazil). Variables analyzed in the study were patients age and sex, stage at diagnosis, site of metastases, Ki-67 (<2, 2–20, >20), Eastern Co-operative of Oncology Group (ECOG) performance status (asymptomatic, symptomatic fully ambulatory, symptomatic in bed less than 50%, symptomatic in bed greater than 50%, and bedridden), current resectability, and current line of therapy, and current therapy (chemotherapy, biological, targeted therapy).

### 2.3. Statistical analysis

The present cross sectional study is of descriptive nature and no statistical hypotheses were tested. All analyses were performed using SAS 9.4 (SAS Institute, Cary).

## 3. Results

### 3.1. Clinical characteristics of the study cohort

The present analysis included a total of 662 patients with pNETs from 9 different countries worldwide (Germany, France, Spain, UK, Japan, China, South Korea, Brazil, Mexico) who received antitumoral therapies. The mean age of the study cohort was 62 years (SD 12 years). 46.1% of pNET patients were female. The majority of patients had an ECOG 1 performance status (63.3%), while 26.0% and 8.6% of patients had an ECOG 0 or 2 status, respectively. 29.5 % of patients were included at dedicated cancer hospitals, 52% at cancer units of hospitals, 11.8% at other units than cancers units of hospitals and 6.8 % of patients by office based practitioners (Table 1).

**Table 1:** Baseline characteristics of study patients.

Variable	Total number of patients (%)
N	662
Age (mean, SD)	62.0 (12.0)
Age group	
16–30	6 (0.9)
31–40	20 (3.0)
41–50	90 (13.6)
51–60	152 (23.0)
61–70	234 (35.3)
71–80	132 (19.9)
>80	28 (4.2)
Women	305 (46.1)
Men	357 (53.9)
Country	
Germany	91 (13.8)
France	145 (21.9)
Spain	95 (14.4)
UK	190 (28.7)
Japan	21 (3.2)
China	38 (5.7)
South Korea	29 (4.4)
Brazil	28 (4.2)
Mexico	25 (3.8)
Stage at diagnosis	
I	10 (1.5)
II	56 (8.5)
III	33 (5.0)
IV	563 (85.0)
Site of metastases (n = 563 in fourth stage)	
Liver	501 (89.0)
Peritoneum	45 (8.0)
Lung	62 (11.0)
Bones	34 (6.0)
Lymph nodes	159 (28.2)
Ki-67 (documented for n = 479)	
<2	46 (9.6)
2–20	324 (67.6)
>20	109 (22.8)
ECOG performance status	
0-asymptomatic	175 (26.4)
1-symptomatic fully ambulatory	419 (63.3)
2-symptomatic in bed less than 50%	57 (8.6)
3-symptomatic in bed greater than 50%	11 (1.7)
4-bedridden	0
Current resectability status (documented for n = 641)	
Inoperable	597 (93.1)
Operable	25 (3.9)
Potentially operable	19 (3.0)
Current line of therapy	
First line	464 (70.1)
Second line	114 (17.2)
Third line	32 (4.8)
Fourth line	9 (1.4)
Adjuvant	31 (4.7)
Neoadjuvant	9 (1.4)
Early stage/primary therapy	3 (0.5)
Current therapy	
Chemotherapy	177 (26.8)
Biological therapy	261 (39.4)
Targeted therapy	208 (31.4)
Other therapies and combinations	16 (2.4)
Inclusion site	
Dedicated cancer hospital	195 (29.5)
Cancer units of an hospital	344 (52.0)
Other units than cancers units of an hospital	78 (11.8)
Office-based practitioner	45 (6.8)

ECOG = Eastern co-operative of Oncology Group.

### 3.2. Tumor characteristics of pNET patients

The most common Union international contre le cancer (UICC) tumor stage was stage IV (85%). UICC stages I, II, and III pNET was observed in 1.5%, 8.5%, and 5% of patients, respectively. With respect to the Ki-67 expression status, 9.6% of patients had a KI-67 expression below 2%. 67.6% of pNET patients had a Ki-67 expression between 2 and 20% and 22.8% of patients showed an expression above 20%. When looking at UICC stage IV patients (n = 563) only, liver metastases (89.0%) represented the most common site of extra-pancreatic tumor manifestation followed by the lymph node metastases (28.2%), lung metastases (11.0%), peritoneal metastases (8.0%), and bone metastases (6.0%). 93.1% of patients were classified as inoperable, 3.9% as an operable and 3.0% of patients as potentially operable (Table 1).

### 3.3. Treatment paradigms of pNET patients

Most patients (70.1%) received systemic antitumoral therapy for pNET in the first-line setting, while 17.2% of pNET patients received second-line treatment. Third (4.8%) and fourth-line (1.4%) treatment as well as neo-adjuvant therapy (1.4%) were less common. 4.7% of pNET patients received anti-tumoral therapy in an adjuvant setting (Table 1). Considering the administered substance class, biological therapy (including somatostatin analogue) was applied to 39.4% of patients, followed by targeted therapies (31.4%, including Everolimus, Sunitinib, …) and cytotoxic chemotherapy.

## 4. Discussion

Gastrointestinal (nonpancreatic) neuroendocrine neoplasia represent a rare but in recent years more frequent tumor entity.^[9]^ Similarly the incidence of pancreatic neuroendocrine neoplasia has constantly risen over time. Nevertheless, global differences in the availability of diagnostic and treatment modalities might have biased data on the epidemiology of these tumors worldwide.^[10]^ In the present study, we analyzed clinical and histopathological characteristics of patients that were diagnosed with a pNET in 9 different countries worldwide (Germany, France, Spain, UK, Japan, China, South Korea, Brazil, and Mexico). By studying a total of 662 patients, we show that the vast majority of patients diagnosed with pNET displayed well or moderate differentiated tumors. Up to 90% of patients identified in our database presented in an advanced disease stage (UICC stage IV) and received systemic therapy. Of note, 93.9 % of patients received systemic antitumoral therapy in palliative intention, while treatment was administered in 1.4% of cases in neoadjuvant and in 4.7% of cases in in an adjuvant setting. Biological therapy was applied to 39.4% of patients, followed by targeted therapies (31.4%) and chemotherapy, highlighting the complexity in the treatment of patients with pNET.

Prognosis and biological behavior of pNET depends on the specific tumor’s growth rate (Ki-67) and histology.^[11]^ Within the 662 patients analyzed here, 77.2% of pNET patients had a Ki-67 expression between 0 and 20%, which is in line to previous data. However, it must be borne in mind that these data may be distorted by regional treatment structures. For example, many G3 patients in Germany are treated by oncologists, while patients with well-differentiated tumors tend to be treated by gastroenterologists or endocrinologists, who are not adequately covered by the IQVIA’s OD database^[5–7]^. Today, data high-grade neuroendocrine neoplasia including NET G3 and NEC are still scarce^[12]^. In a recent analysis including 1513 NEN only 130 were classified as NETs G3 (9%). In our analyses, about 20% of patients displayed a Ki-67 above 20%, nevertheless we are unable to further subdivide them according to their histology. The rarity of NET G3 explains the fact that at up to now no data from randomized trials on the optimal treatment of this entity are available.^[10]^ However, there is consensus that cytotoxic chemotherapy should be administered. In our analyses 26.8% of patients received cytotoxic chemotherapy which is in good accordance to the number of patients with elevated Ki-67 (>20%). Unfortunately, we have no access on data regarding the specific substances used in the individual patients. However, it seems likely that most patients will have been treated with classical cytotoxic agents including cisplatin, etoposide, 5-FU, temozolomide or streptozotocin as recommended by current guidelines^[10]^. Nevertheless, the prognosis of patients with G3 NET/ NEC is much worse than that of patients with well differentiated NET (G1/G2). Thus, our data underline the fact that in most patients the overall prognosis is rather favorable despite featuring a metastasized disease stage.

We have recently demonstrated important variations in bot clinical and pathological characteristics of patients with nonpancreatic gastrointestinal NET among 5 European countries.^[4]^ Here we extended this previous analysis by specifically examining patients with pNET and including additional countries. Among the total of 662 pNET patients that were part of our present analysis, 91 (13.8 %) were derived from Germany, 145 (21.9 %) from France, 95 (14.4 %) from Spain and 190 (28.7 %) from the UK. In addition, 21 (3.2 %) patients from Japan, 38 (5.7%) from China, 29 (4.4 %) from South Korea, 28 (4.2 %) from Brazil, and 25 (3.8%) from Mexico were included into the analysis. As pointed out, pNEN represent very rare diseases with an annual incidence of 0.48/100,000.^[13,14]^ Previous studies on the clinical characteristics of pNET patients have often failed due to difficulties in recruiting sufficient patients for investigations. Against this background, the large number of patients included and the large number of countries considered represent a major strength of our study. Nevertheless, the patient numbers studied are too small to statistically meaningfully investigate specific differences between different geographic regions or countries. Of note, our study faces important limitations. Some of these limitations are specific to NEN, others of which reflect general imitations of the database as recently described.^[4]^ Regarding NEN, data on a potential hereditary background are lacking. Moreover, no data on the functional activity are available. Finally, it is impossible to fully exclude that NEC G3 have been misclassified as NET G3. In addition, due to the study design and the fact that most patients were included by oncologists, patients with localized disease stages might be less likely to be included into the study (Table 1). Thus, it seems possible that the database might not be representative for the whole spectrum of pNEN. Regarding the database, it is important to note that only drug-treated patients were included and that the original questionnaire was not designed for the concrete research purpose. Missing variables such as genetic factors and socioeconomic status are further limitations. Finally, no causal relationships but only associations can be estimated in studies like this and comparison with other established data bases are lacking. Nevertheless, the database was used for many different studies and has demonstrated it suitability for research purpose in many different clinical analyses.^[8]^

In summary, our data highlight that pancreatic neuroendocrine neoplasia are diagnosed in advanced tumor stages, globally. Systemic treatment was the most commonly used treatment modality. Despite being descriptive only and potentially not reflecting the characteristics of all patients with NET, such data may help to better understand the specific epidemiology of pNET worldwide and should trigger further epidemiological research allowing to better understand the pathophysiology of pancreatic NEN and to optimize the management of patients with these tumors.

## Author contributions

CR, SHL, and KK, KK performed statistical analyses and generated figures and tables. CR, SHL, HJ and KK wrote the manuscript, JE, SK, AK, HJ provided intellectual input, all authors agreed to the final version of the manuscript.

**Conceptualization:** Sven H. Loosen, Karel Kostev.

**Data curation:** Karel Kostev.

**Formal analysis:** Karel Kostev.

**Project administration:** Sven H. Loosen.

**Supervision:** Karel Kostev.

**Validation:** Sven H. Loosen, Karel Kostev.

**Visualization:** Sven H. Loosen.

**Writing—original draft:** Sven H. Loosen, Karel Kostev, Johannes Eschrich, Sarah Krieg, Andreas Krieg.

**Writing—review and editing:** Sven H. Loosen, Karel Kostev, Johannes Eschrich, Sarah Krieg, Andreas Krieg.
